# Time course of eccentric hamstring strength recovery after a competitive match in under-20-year-old football players

**DOI:** 10.1016/j.jesf.2026.200523

**Published:** 2026-09-09

**Authors:** Roberto Carlos Rebolledo-Cobos, Jheifer Páez-Almentero, Carlos Rolong-Donado, Bruno Manfredini Baroni

**Affiliations:** a Graduate Program in Rehabilitation Sciences, Federal University of Health Sciences of Porto Alegre (UFCSPA), Porto Alegre, RS, Brazil; b Faculty of Health, Exact and Natural Sciences, Universidad Libre, Barranquilla, Colombia; c Artificial Intelligence and Robotics Research Center, Universidad Simón Bolívar, Barranquilla, Colombia; d Department of Sports Medicine, Junior de Barranquilla Football Club, Barranquilla, Colombia

**Keywords:** Soccer, Nordic hamstring exercise, Fatigue, Injury prevention

## Abstract

**Background/objective:**

Elite football teams often compete under congested schedules with less than 72 h between matches; however, whether this period is sufficient for eccentric hamstring strength recovery remains unclear. This study aimed to investigate the time course of eccentric hamstring strength recovery following an official football match in elite male under-20-year-old players.

**Methods:**

Twenty outfield players (18 ± 1 years) from two football academies who completed ≥80 min in the first official match of the season were assessed one day pre-match (baseline) and approximately 24, 48, and 72 h post-match (MD+1, MD+2, and MD+3). Peak eccentric hamstring strength was simultaneously assessed in both limbs while performing the Nordic hamstring exercise. The assessment's relative minimal detectable change (MDC; 15.9%) was used to assess individual responses; players were classified as *‘not recovered’* when the percentage strength reduction from baseline was equal to or exceeding this threshold.

**Results:**

When data from both limbs were pooled, eccentric hamstring strength was significantly lower (p < 0.05) than baseline at MD+1 (16 ± 10% reduction) and MD+2 (13 ± 9% reduction), but not at MD+3 (8 ± 9% reduction). Conversely, individual response analysis revealed that 40% of players still had at least one limb classified as not recovered at MD+3.

**Conclusion:**

Eccentric hamstring strength returned to baseline at the group level within 72 h after a competitive match, but individual recovery remained heterogeneous. These findings support the use of individualized monitoring to optimize load management in elite youth football.

## 1 Introduction

Hamstring strain injury (HSI) is consistently reported as one of the most prevalent injuries across various levels of football (soccer). A review including 13 studies and 3868 players with two million hours of sport exposure reported that HSIs accounted for 4% to 13% of all football injuries.^1^ Elite senior and under-20-year-old (U20) teams can expect approximately five to eight time-loss HSIs per season in a 25-player squad.^2^^,^^3^ Most HSIs are classified as mild to moderate in severity and typically result in up to four weeks of time lost from sport.^4^ However, more severe HSIs, particularly those involving intramuscular tendon damage, may require extended recovery periods of two to three months or longer.^5^ In addition to representing a performance loss and an economic burden for clubs,^6^ injuries may reduce young players’ opportunities to progress to the professional level.^7^ Therefore, effective prevention strategies are particularly relevant in football youth academies.

Within the multifactorial risk profile of HSIs, lower eccentric knee flexor strength has been identified as a potentially modifiable neuromuscular factor.^8^^,^^9^ Prospective cohort studies have reported that lower preseason eccentric hamstring strength may be associated with a greater likelihood of subsequent HSI in footballers, particularly when assessed using isokinetic dynamometry or the Nordic hamstring exercise (NHE).^10^^,^^11^ Nevertheless, the clinical predictive value of isolated preseason strength assessments remains limited. Although lower eccentric knee flexor strength has been associated with subsequent hamstring strain injury in some cohorts, these associations are generally weak and may not adequately distinguish players who will sustain an injury from those who will remain uninjured.^12^^,^^13^ Moreover, studies in professional football have shown that isolated or comprehensive strength-testing protocols have poor clinical value for predicting subsequent lower-extremity or hamstring injuries.^14^^,^^15^ Accordingly, eccentric knee flexor strength should not be interpreted as a standalone screening tool, but rather as one relevant neuromuscular factor within the multifactorial and dynamic profile of hamstring strain injury risk.

In addition, the predictive value of isolated preseason strength assessments may be limited by the dynamic nature of muscle strength, which can fluctuate over short periods in response to training load, match exposure, fatigue, recovery status, and other contextual factors.^16^ Therefore, rather than relying solely on preseason strength testing to estimate the risk of future HSIs,^13^ systematic monitoring of athletes’ strength throughout the competitive period may provide more clinically meaningful information for prevention purposes. For instance, Wollin et al.^17^ and Adams et al.^18^ conducted strength testing within two days after a match in male youth and professional football players, respectively. Subsequent individualized training-load adjustments for players presenting hamstring strength impairments were associated with lower HSI incidence and time loss, supporting the potential value of strength monitoring as a prevention approach.

Despite the International Olympic Committee's recommendation of at least 96 h between matches to ensure adequate recovery, congested fixture schedules involving up to three matches per week remain common in contemporary football.^19^ These congested schedules have been associated with a higher incidence of muscle injuries than fixtures separated by six or more days.^20^ Considering the conceptual framework proposed by Ekstrand et al.,^9^ which suggests that players who start a match with a strength deficit (e.g., due to residual fatigue) may be at greater risk of HSI during the match, increasing attention has been directed toward understanding the time course of muscle strength recovery following football matches. Several studies have reported that group mean hamstring isometric strength can return to baseline values within 48 to 72 h after match play.^21^, ^22^, ^23^, ^24^, ^25^ However, evidence on the recovery kinetics of eccentric hamstring strength remains limited. Rhodes et al.^26^ assessed professional football players after a simulated football-specific fatigue protocol using isokinetic dynamometry and reported eccentric torque reductions that remained evident throughout the 72-h monitoring period. Bueno et al.^27^ assessed professional football players after a preseason friendly match using an instrumented NHE and found significant strength reductions at 48 h. Although group mean values returned to baseline by 72 h, individual response analysis showed that some players had not fully recovered at this time point. Therefore, the limited number of available studies and the inconsistency of findings across them perpetuate uncertainty regarding the time course required for full recovery of eccentric hamstring strength following football matches.

Although congested schedules are more frequently observed in senior football teams, similar scenarios may also occur in youth academies. A prominent example is the São Paulo Youth Cup, one of the largest U20 tournaments worldwide, in which teams may compete with intervals of less than 72 h between matches.^28^ Against this background, the present study extends the available evidence by examining elite male U20 players after an official competitive match and by integrating group-level, individual, and limb-specific analyses of eccentric knee flexor strength recovery. Therefore, the aim of this study was to investigate the time course of eccentric hamstring strength recovery following an official football match in elite male U20 players. As a secondary objective, the study examined individual variability in recovery kinetics.

## 2 Methods

### 2.1 Experimental approach to the problem

A longitudinal observational study with repeated measures was conducted involving two elite football youth academies based in Barranquilla (Colombia) as they competed in the first official match of the 2024 season. Eccentric hamstring strength assessments were carried out at four distinct time-points: one day before the match (Baseline, on Saturday), one day after the match (MD+1, on Monday), two days after the match (MD+2, on Tuesday), and three days after the match (MD+3, on Wednesday). The competitive match was played between 7:30 and 9:20 a.m., and all assessments were conducted within the same morning time window (7:00–8:00 a.m.) to minimize potential variability related to time of day. All four assessments were conducted before any scheduled team training session. The baseline testing was performed immediately before a low-intensity technical training session, as typically scheduled on the day before a match. The MD+1 testing occurred prior to a 60-min regeneration session, which consisted of low-intensity aerobic activities prescribed by the clubs' technical staff. The MD+2 testing was performed on a designated rest day without training. The MD+3 testing took place immediately before the squad's next scheduled full training session.

### 2.2 Subjects

Male football players aged between 17 and 20 years, formally registered with their clubs and competing in the U20 team, were enrolled in this study. To be eligible, players were required to be free from musculoskeletal injury or physical discomfort during the preseason and data collection period. In addition, according to the clubs’ medical records, players with a history of HSI during the previous season were not included. In agreement with the technical and medical staff of the clubs, only outfield players who were expected to tolerate near-complete match exposure were considered for participation. Therefore, from a combined roster of 38 athletes, the analysis was restricted to outfield players who completed at least 80 min of the match, as this threshold was used to represent substantial exposure to the physical demands of a real competitive match. Goalkeepers were not assessed due to their distinct match demands.

All participants were engaged in five-week preseason training programs designed and supervised by the respective coaching staffs of each academy. During this period, players completed five to six morning field-based training sessions per week (80–100 min each) and two weekly evening strength-training sessions. In line with the routine preseason practices implemented by the participating clubs, no specific eccentric knee flexor exercises were prescribed. This decision reflected the clubs' standard training approach rather than a methodological requirement of the study. During the preseason period, training participation, effort tolerance, and any episodes of musculoskeletal discomfort or injury potentially affecting maximal strength performance were monitored and recorded by the clubs’ technical and medical staff; players presenting symptoms or events that could limit maximal testing were not included in the study. The final two training sessions preceding the baseline assessment were deliberately prescribed at low volume and intensity, ensuring that all players completed at least 48 h free from strenuous training before testing, thereby minimizing the potential influence of residual fatigue.

All participants or their guardians provided informed consent before data collection commenced. The study was approved by the 575 Universidad Metropolitana Ethics Committee.

### 2.3 Eccentric hamstring strength assessment

**Fig. 1:**
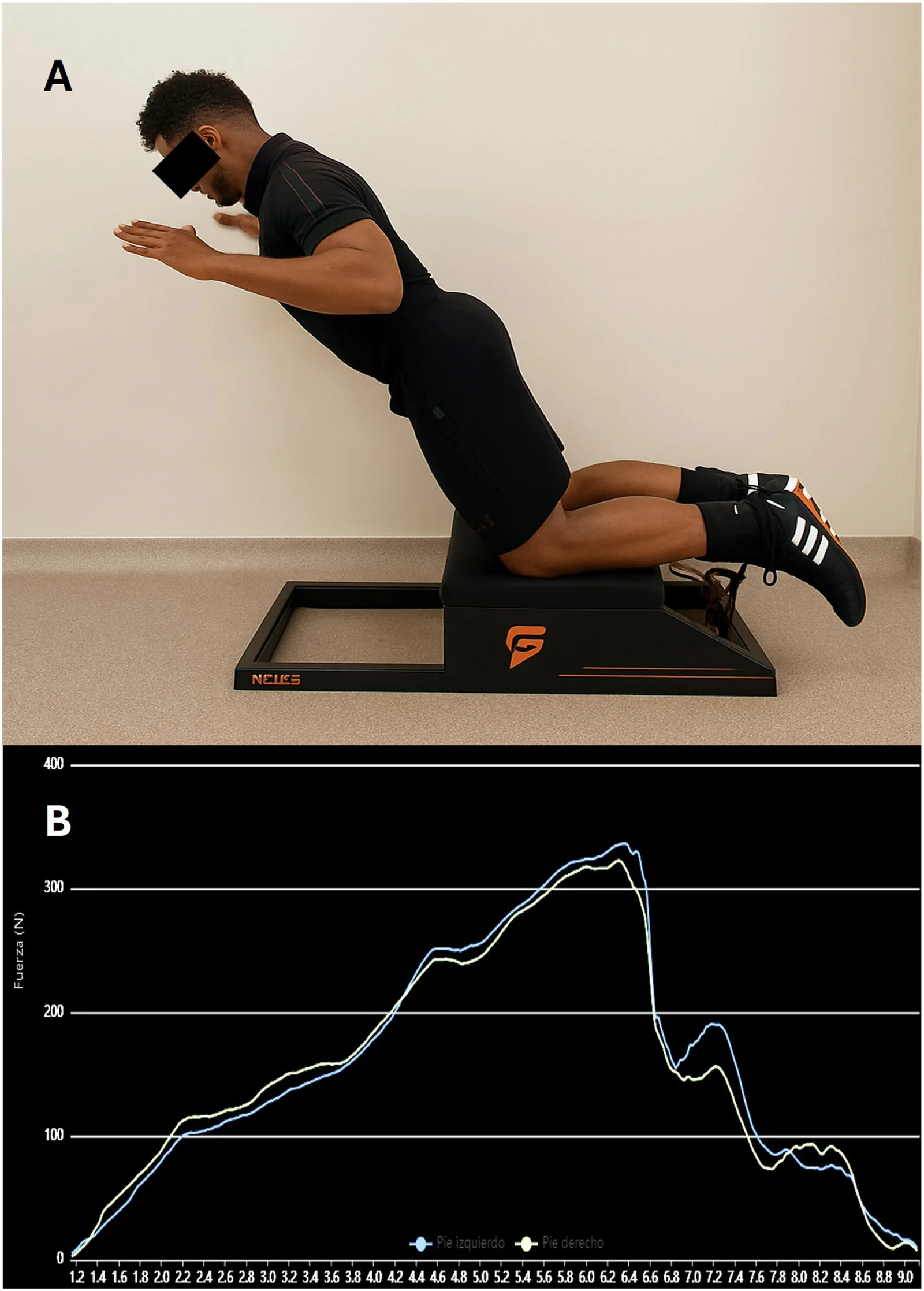
Evaluation of eccentric hamstring strength during the execution of the Nordic hamstring exercise (A). Real-time force curves captured by custom-made device (B).

The force generated by the knee flexor muscles during the execution of the NHE was assessed using real-time recordings obtained from a custom-made device developed specifically for this study (Fig. 1). The device stems from the prototype validated by Opar et al.^29^ and has been gaining widespread adoption globally.^14^^,^^30^ This tool integrates two S-type load cells, each with a maximum capacity of 980 N and an accuracy of 0.02 N. These load cells are connected to data acquisition cards featuring 24-bit analog-to-digital converters with a sampling rate of 80 samples per second, integrated into a 32-bit single-board computer. Data transmission is carried out wirelessly via Bluetooth Low Energy to dedicated software, enabling real-time monitoring of the tension force exerted by each limb during the exercise. The system generates a synchronized array consisting of three data points: force from the left lower limb, force from the right lower limb, and the corresponding timestamp.

Before testing, participants performed a standardized general warm-up designed and overseen by the team's strength and conditioning coach. Every assessment was subsequently conducted by the same two investigators, both of whom were highly experienced with the procedures. Participants commenced the test kneeling on a padded platform with hips extended and trunk upright; they were instructed to lower the torso toward the floor at a constant velocity until failure (at least 3 s per repetition), keeping the hips and spine aligned and the elbows flexed so that the palms faced forward at shoulder height. Participants were instructed to use their upper limbs to absorb the fall and to return to the initial position. Each player performed three repetitions separated by 15 s of rest, following the protocol adopted by Bueno et al.^27^ This inter-repetition interval was standardized across all assessments and selected to ensure methodological consistency with the most comparable study while maintaining logistical feasibility in the applied field setting, considering the large number of players from two clubs, the availability of a single testing device, and the limited testing window allocated by the clubs. Although a short recovery interval may have allowed some degree of within-test fatigue, inspection of the testing records showed that, in more than 70% of the assessments, the highest peak eccentric force was achieved during the second or third repetition.

Although the device records synchronized force-time data for each limb during the NHE, only peak force was retained for analysis. Peak force was selected as the primary outcome because it represents the main force-based variable derived from instrumented NHE devices and has demonstrated acceptable test–retest and between-session reliability in field-based football settings.^27^^,^^29^^,^^31^ In addition, peak force has been used to characterize eccentric knee flexor strength in large samples of senior and U20 football players assessed with load-cell devices.^32^ Because the present analysis focused on within-player changes relative to each participant's own baseline, potential between-player differences related to body size were not the primary focus of interpretation.

Test–retest reliability of the custom-made device was established before the present study in twenty-one U20 football players from the same clubs, during the initial preseason period, with no musculoskeletal discomfort or limitations affecting maximal strength testing. The test and retest sessions were conducted 48 h apart under the same testing conditions, including the standardized warm-up, testing procedures, assessors, number of repetitions, inter-repetition rest interval, and device configuration used in the main study. For each limb, peak force was defined as the highest value obtained across the three repetitions. Reliability was assessed using a two-way mixed-effects, single-measure intraclass correlation coefficient, ICC (3,1), with a 95% confidence interval. The results showed good test–retest reliability: initial test = 367.1 N (95% CI 349.8–384.5), retest = 359.5 N (95% CI 344.5–374.6), ICC (3,1) = 0.85 (95% CI 0.71–0.92), typical error = 21.03 N, and coefficient of variation = 5.8%. The minimal detectable change at the 95% confidence level (MDC95) was calculated as the typical error × 1.96 × √2. The resulting absolute MDC was subsequently expressed as a percentage of the initial test mean to obtain the relative MDC used for the individual recovery classification, corresponding to 58.3 N or %. This relative MDC was subsequently used to classify limb-specific post-match changes as recovered (<15.9%) or not recovered (≥15.9%).

### 2.4 Statistical analysis

Two separate two-way repeated-measures ANOVAs were conducted to examine changes in eccentric hamstring strength: one using absolute strength values and the other using percentage changes relative to baseline. In both models, time (baseline, MD+1, MD+2, and MD+3) and limb (dominant and non-dominant) were entered as within-subject factors. Bonferroni-adjusted post-hoc comparisons were performed following significant main effects. Statistical significance was set at p < 0.05. Effect sizes between baseline and post-match measures were calculated using Cohen's d, and effect sizes were interpreted as trivial (d < 0.2), small (d > 0.2), medium (d > 0.5), or large (d > 0.8).

To assess individual responsiveness, the device's MDC was expressed in relative terms, allowing post-match strength changes to be interpreted as a percentage of each player's baseline performance. Players were classified as ‘recovered’ when the percentage strength reduction was < 15.9% (the device's relative MDC) and as ‘not recovered’ when the reduction was equal to or exceeding this threshold. Expressing the MDC in relative terms facilitated the interpretation and visualization of individual recovery responses and was consistent with the analytical approach adopted in the closest previous study.^25^

## 3 Results

Twenty outfield players (the entire starting line-ups from the two participating teams, with goalkeepers excluded) were enrolled: age, 17.9 ± 0.9 years; height, 1.78 ± 0.06 m; body mass, 74.0 ± 6.7 kg; body-fat, 12.4 ± 3.5%. No players reported a history of HSI in the previous season. Although each team made three substitutions during the match, all substituted players retained in the analysis completed more than 80 min of playing time.

**Fig. 2:**
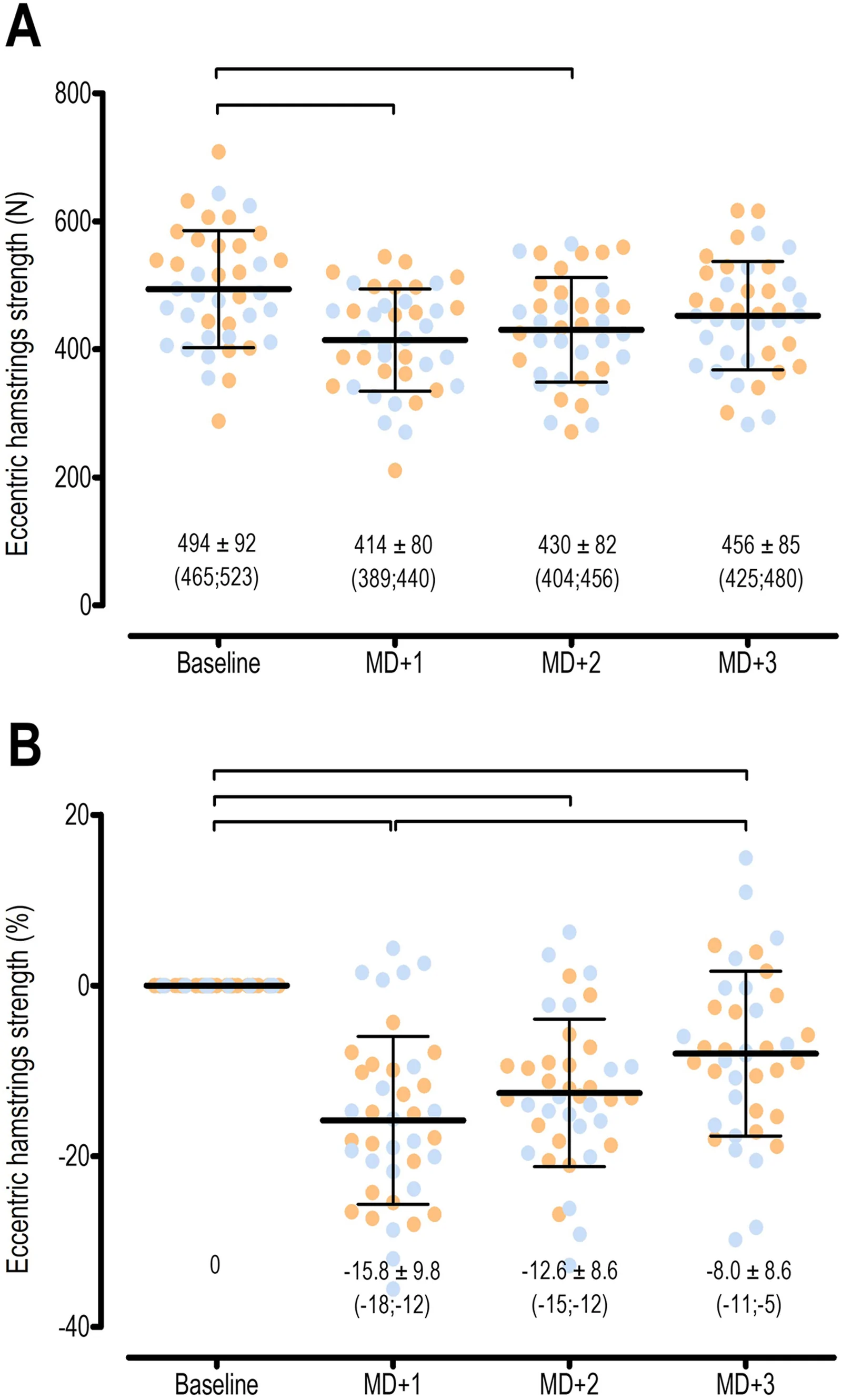
Absolute eccentric knee flexor strength (A) and percent change from baseline (B). Individual data points are shown for both limbs at each assessment time point: orange dots represent dominant limbs and blue dots represent non-dominant limbs. Values below each time point are presented as mean ± standard deviation, with 95% confidence intervals in parentheses. Horizontal lines indicate statistically significant pairwise differences between time points (p < 0.05).

There was no significant time × limb interaction for either absolute or baseline-relative eccentric hamstring strength (Table 1). Therefore, data from the dominant and non-dominant limbs were pooled to interpret the significant time effect in both absolute and relative terms (Table 1). Fig. 2-A shows the temporal changes in eccentric hamstring strength across the post-match recovery period: baseline strength values were significantly higher than those recorded at MD+1 (p < 0.001; effect size = 0.9; effect size 95% CI 0.3–1.5) and MD+2 (p = 0.005; effect size = 0.8; effect size 95% CI 0.2–1.3), whereas no significant difference was observed between baseline and MD+3 (p = 0.171; effect size = 0.5; effect size 95% CI 0.1–1.0). Fig. 2-B shows the percent changes from baseline to post-match timepoints: significant lower values were found at MD+1 (p < 0.001; effect size = 1.9; effect size 95% CI 1.2–2.6), MD+2 (p < 0.001; effect size = 1.5; effect size 95% CI 0.9–2.1) and MD+3 (p < 0.001; effect size = 1.0; effect size 95% CI 0.4–1.6).

**Table 1:** Two-way repeated-measures ANOVA for eccentric hamstring strength.

Source of variation	∑^2^	df	F	p	η^2p^	ω^2^
Absolute values						
Time	1.432	3	6.773	<0.001	0.117	0.096
Limbs	4.363	1	6.191	0.014	0.039	0.028
Limbs x Time	1346	3	0.063	0.979	0.001	0.000
Relative values						
Time	5644	3	27.85	<0.001	0.354	0.339
Limbs	19.62	1	0.290	0.591	0.001	0.000
Time x Limbs	32.02	3	0.167	0.918	0.003	0.000

**Fig. 3:**
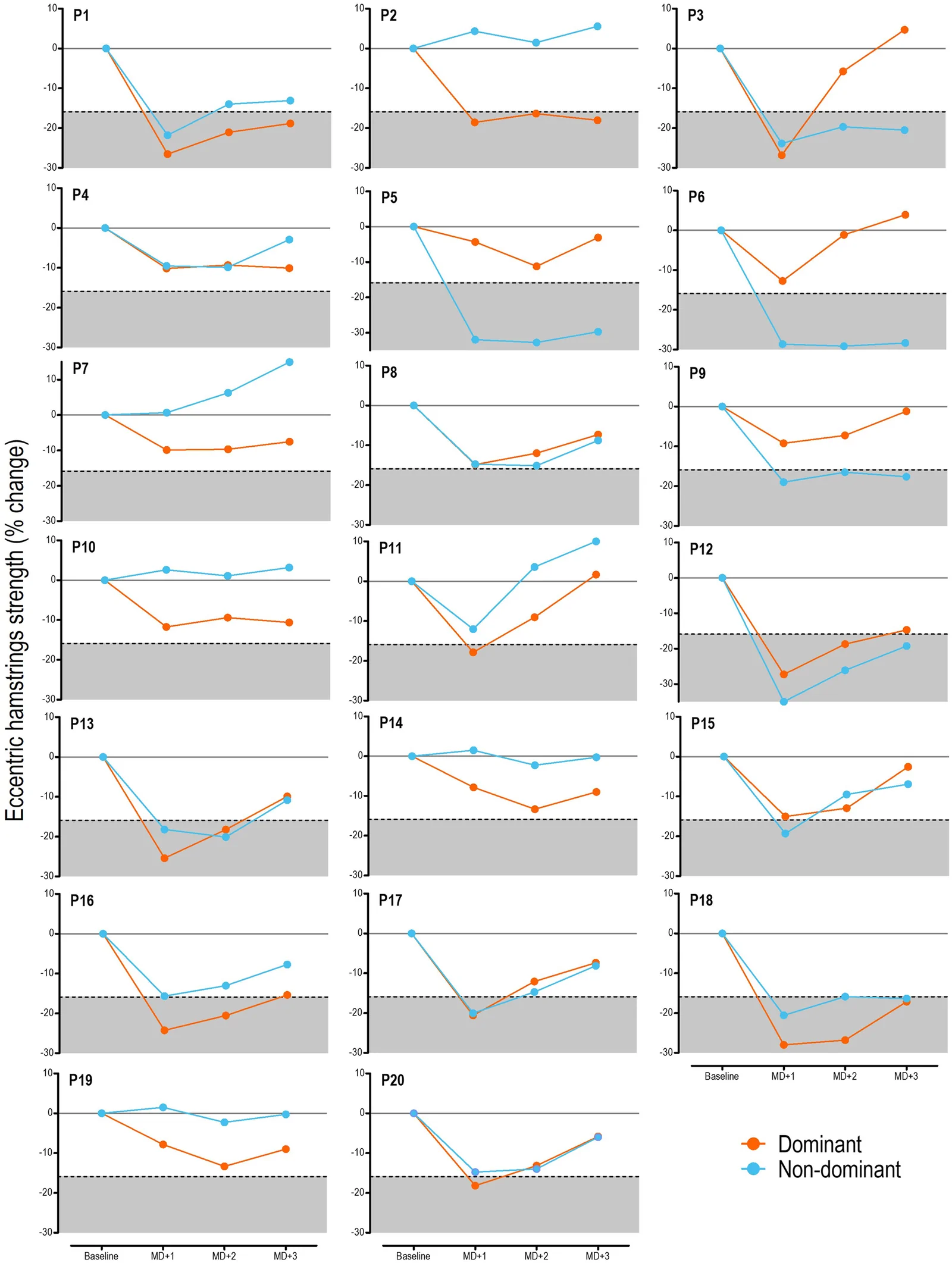
Individual recovery trajectories of dominant (orange) and non-dominant (blue) limbs across post-match days. Values are expressed as percentage change from baseline. Background zones denote recovery status: white = recovered (deficit < 15.9%); grey = not recovered (deficit ≥ 15.9%).

Fig. 3 shows the individual recovery trajectories of the dominant (orange) and non-dominant (blue) limbs, expressed as percentage change from baseline. These profiles highlight substantial inter-individual variability that is obscured when examining group averages. At MD+1, 14 of 20 players (70%) had at least one limb classified as not recovered, as the post-match reduction in eccentric hamstring strength reached or exceeded the MDC threshold (≥15.9%), indicating a widespread loss of eccentric strength. The proportion of players not recovered in at least one limb decreased to 50% at MD+2 and 40% at MD+3. Moreover, Fig. 3 illustrates that the kinetics of strength recovery are not consistent between limbs for some athletes.

## 4 Discussion

To the best of our knowledge, this is the first study to describe the recovery kinetics of hamstring eccentric strength following a football match in U20 players. The main finding was that athletes showed a significant post-match reduction in eccentric hamstring strength over time, with a similar temporal pattern in both limbs during the three-day recovery period. From a group-level perspective, eccentric hamstring strength was restored approximately 72 h post-match. Nevertheless, the individual analysis revealed substantial heterogeneity in recovery responses across players, as well as dissociation in recovery patterns between the dominant and non-dominant limbs in some cases.

Our findings indicate an average reduction in eccentric hamstring strength of approximately 16% at MD+1, 13% at MD+2, and 8% at MD+3. These reductions are greater than those previously reported for isometric hamstring strength, which range from 5 to 13% at MD+1 21, 22, 23, 24, 25 and have been shown to return to baseline within 48 h in some studies.^21^^,^^23^^,^^25^ Therefore, the magnitude of match-induced deficits appears to be dependent on the type of muscle contraction. This is consistent with the findings of Cosio et al.,^22^ who reported that maximal voluntary isometric strength had returned to baseline, whereas rate of torque development remained impaired by MD+3, suggesting distinct recovery kinetics across different strength expressions. Further studies are needed to better characterize the recovery trajectories of different contraction modes following football matches. From a practical standpoint, our findings indicate that post-match recovery may differ according to the mode of muscle contraction assessed. Therefore, isometric and eccentric strength assessments should not necessarily be considered interchangeable when monitoring neuromuscular recovery after competition.

Studies examining the recovery kinetics of eccentric hamstring strength following a football match are scarce.^26^^,^^27^ In the study by Rhodes et al.,^26^ professional players completed a simulated match protocol (the Soccer Aerobic Field Test, SAFT90) and eccentric hamstring strength was assessed using isokinetic dynamometry. The authors reported significant deficits ranging from 12% to 24% across three angular velocities during the three-day post-match monitoring period.^26^ If 72 h is insufficient for post-match recovery, this would have implications for training design and injury management, particularly during fixture-congested periods. The findings of the present study and those reported by Bueno et al.^27^ only partially support this premise. Both studies used the NHE to assess eccentric hamstring strength and demonstrated that squads had already restored baseline force production capacity within 72 h following a real football match. However, individual response analyses from both studies revealed that some players still exhibited strength deficits above the MDC, indicating suboptimal recovery three days after the match.

Given the similarity in experimental design, including the use of a real football match and strength assessment via the NHE, the study by Bueno et al.^27^ provides the most appropriate comparison with our investigation. Similar to our findings, Bueno's study observed significant eccentric hamstring strength reductions up to MD+2, with values returning to baseline by MD+3. However, it should be noted that the average strength loss at MD+2 in U20 players (∼12%) was approximately twice that reported in professional players previously assessed.^27^ This greater magnitude of eccentric strength reduction may be attributable to higher fatigue demands in the official match used in our study compared with the friendly match in the study by Bueno et al. However, a reduced recovery capacity in U20 players relative to professional players cannot be ruled out. In addition, players in the present study completed an official competitive match following a five-week preseason period in which specific eccentric knee flexor training was not systematically included. The limited exposure to eccentric hamstring loading during preseason may have contributed to the magnitude of strength reductions and the recovery time course.

The individual recovery kinetics in response to a given exercise demand, such as sport-specific sprint training^33^ or an actual football match,^21^, ^22^, ^23^, ^24^, ^25^^,^^27^ represent a growing area of interest within the scientific community. This type of analysis alerts practitioners to the relevance of individualized monitoring, particularly when dealing with measures that exhibit high inter-individual variability. Regarding the recovery kinetics of eccentric hamstring strength, Bueno et al.^27^ demonstrated that six out of the 15 athletes monitored in their study (i.e., 40%) did not achieve a full recovery status within 72 h, based on a residual fatigue threshold derived from the coefficient of variation of the measure. In the present study, eight of 20 players (40%) remained classified as not recovered in at least one limb at MD+3, according to the relative MDC threshold. Additionally, by analyzing dominant and non-dominant limbs separately, the present study also highlights that some athletes may exhibit inconsistent recovery trajectories between limbs. Beyond highlighting the heterogeneity of responses, a visual inspection of Fig. 3 (which depicts the individual trajectories of residual fatigue in each athlete's limb) alerts practitioners to the relevance of monitoring of eccentric hamstring strength during post-match recovery.

A theoretical framework by Ekstrand et al.^9^ offers a plausible explanation for why players starting a match with a strength deficit may face a mismatch between their physical capacity and the demands of play. Residual post-match reductions in eccentric hamstring strength may represent one plausible factor contributing to recovery challenges during congested schedules. However, because the present study did not collect match-load data or subsequent injury outcomes, it cannot determine whether these reductions explain the increased incidence of muscle injuries previously reported during congested match periods.^20^ From an applied perspective, the observed residual reductions in eccentric knee flexor strength may be relevant when planning subsequent training exposure or match participation, particularly for players whose reductions exceed the MDC threshold. However, the present findings do not establish a specific strength threshold for restricting training or match participation, nor do they demonstrate that modifying exposure on the basis of eccentric strength changes prevents subsequent HSI.

Monitoring reductions in eccentric strength in both limbs through hamstring strength testing may represent a promising strategy to support HSI prevention programs in football clubs. To our knowledge, no trials have specifically monitored eccentric hamstring strength during post-match recovery as a preventive measure. However, Wollin et al.^17^ and Adams et al.^18^ assessed isometric hamstring strength on MD+2 and implemented individualized training load adjustments for players presenting strength deficits. This approach led to an approximately 80% reduction in HSI incidence compared with previous seasons without strength monitoring in young players,^17^ and a 20% decrease in HSI burden among professional footballers.^18^ Although post-match isometric strength monitoring combined with individualized load-management strategies has been associated with lower HSI burden in previous football settings,^17^^,^^18^ it remains unknown whether an equivalent approach based on eccentric hamstring strength monitoring would improve injury-related outcomes. Prospective intervention studies are required to determine whether monitoring-guided adjustments based on post-match eccentric strength reductions can reduce HSI incidence or time loss. Nonetheless, it is important to note that maximal eccentric testing with the NHE may impose greater stress on athletes than isometric testing, warranting careful integration into the team's training routine.

Some limitations of this study should be acknowledged. First, the lack of information regarding match demands, including both internal and external workload measures, limits the ability to establish dose–response relationships between match load and eccentric hamstring strength recovery. Second, although the clubs’ medical records were reviewed to identify HSI history during the previous season, information on injuries sustained before players joined their current clubs or during earlier age categories could not be obtained. Third, post-match factors (such as sleep quality and duration, hydration status, diet, and lifestyle habits) were not assessed and the absence of additional recovery markers (such as perceptual scales, biochemical indicators, and other performance tests) may have limited the depth of discussion regarding eccentric strength data. Consequently, although the present study describes the temporal pattern of post-match eccentric hamstring strength recovery, it cannot explain the determinants of inter-individual variability in recovery responses. Fourth, the omission of specific eccentric hamstring strength training during the preseason reflects usual practice in the applied setting studied. This should be considered a limitation, as preseason NHE training could have influenced baseline strength levels and subsequent responses to match-play fatigue. Therefore, the present findings should be interpreted in the context of players who were not systematically exposed to this training stimulus. Fifth, although some players from the participating clubs were exposed to the NHE during the device reliability procedures and as part of the clubs' routine preseason assessment, no specific familiarization session with the NHE was conducted immediately before the present data collection protocol. Lastly, although the number of players assessed in this study is greater than in previous reports,^21^, ^22^, ^23^, ^24^, ^25^^,^^27^^,^^33^ this should not be considered a large-sample study.

Conversely, several strengths of the study should also be highlighted. The football-specific fatigue was induced ecologically, through an actual match between two teams with strong local rivalry rather than a simulated protocol, enhancing the external validity of our findings. Additionally, the use of a customized, low-cost measurement instrument with test–retest reproducibility comparable to the gold standard may encourage sports teams to adopt similar approaches for monitoring eccentric hamstring strength through a quick and feasible test suitable for real-world football youth academies.

## 5 Conclusion

Elite U20 football players sustained significant eccentric hamstring strength deficits in the first two days after a competitive match. From a group-level perspective, eccentric hamstring strength was restored approximately 72 h post-match. However, individual analyses suggest that this time window was not sufficient for full strength recovery in 40% of the players evaluated in this study conducted in a real-world football academy setting. These findings support the potential value of individualized eccentric hamstring strength monitoring for informing post-match recovery and load-management decisions.

## Patient’s privacy and consent for publication

All participants or their guardians provided informed consent before data collection commenced.

## Ethical approval statement

The study was approved by the 575 Universidad Metropolitana Ethics Committee.

## Authors contributions

Roberto Rebolledo-Cobos: Conceptualization, Methodology, Investigation; Jheifer Páez-Almentero: Data curation, Visualization, Investigation; Carlos Rolong-Donado: Data curation, Visualization, Investigation; Bruno Manfredini Baroni: Writing- Reviewing and Editing.

## Conflict of interest

The authors declare that they have no known competing financial interests or personal relationships that could have appeared to influence the work reported in this paper.
